# Identifying an optimal machine learning model generated circulating biomarker to predict chronic postoperative pain in patients undergoing hepatectomy

**DOI:** 10.3389/fsurg.2022.1068321

**Published:** 2023-01-06

**Authors:** Ying Hong, Yue Li, Mao Ye, Siyu Yan, Wei Yang, Chunling Jiang

**Affiliations:** ^1^ Department of Anesthesiology, West China Hospital, Sichuan University and The Research Units of West China (2018RU012), Chinese Academy of Medical Sciences, Chengdu, China; ^2^ Laboratory of Anesthesia and Critical Care Medicine, Department of Anesthesiology, Translational Neuroscience Center, West China Hospital, Sichuan University, Chengdu, China

**Keywords:** chronic pain, post-hepatectomy, machine learning (ML), SVM, prediction model

## Abstract

Chronic postsurgical pain (CPSP) after hepatectomy is highly prevalent and challenging to treat. Several risk factors have been unmasked for CPSP after hepatectomy, such as acute postoperative pain. The current secondary analysis of a clinical study sought to extend previous research by investigating more clinical variables and inflammatory biomarkers as risk factors for CPSP after hepatectomy and sifting those strongly related to CPSP to build a reliable machine learning model to predict CPSP occurring. Participants included 91 adults undergoing hepatectomy who was followed 3 months postoperatively. Twenty-four hours after surgery, participants completed numerical rating scale (NRS) grading and blood sample collecting. Three months after surgery, participants also reported whether CPSP occurred through follow-up. The Random Forest and Support Vector Machine models were conducted to predict pain outcomes 3 months after surgery. The results showed that the SVM model had better performance in predicting CPSP which consists of acute postoperative pain (evaluated by NRS) and matrix metalloprotease 3 (MMP3) level. What's more, besides traditional cytokines, several novel inflammatory biomarkers like C-X-C motif chemokine ligand 10 (CXCL10) and MMP2 levels were found to be closely related to CPSP and a novel spectrum of inflammatory biomarkers was created. These findings demonstrate that the SVM model consisting of acute postoperative pain and MMP3 level predicts greater chronic pain intensity 3 months after hepatectomy and with this model, intervention administration before CPSP occurs may prevent or minimize CPSP intensity successfully.

## Introduction

Chronic post-surgery pain after hepatectomy remains a daunting challenge, casting a shadow over the patient's heart. Approximately 40% of post-hepatectomy patients suffered from unbearable CPSP (1), for those after liver transplantation, the incidence of CPSP was surprisingly up to 70.5% (2). Pain itself may not immediately bring threats toward life; people continue to live with their pain. But it strongly disturbs post-operative rehabilitation and post-discharge life. Clinically significant chronic pain often raises diverse mental symptoms, including mood disturbances (28%), sleep disturbances (30%), and reduced enjoyment of life (30%) (3). It is, therefore, crucial to identify risk factors and models that can predict CPSP and intervene before it occurs.

CPSP is deﬁned as pain that persists past normal healing time (4), usually lasting or recurring longer than 3 months (5). The mechanisms lying under CPSP might relate to the whole pain progress. It is now well established that locally inflammatory mediators following tissue injury can not only cause pain by binding to their receptors on nociceptive primary sensory neurons (nociceptors) but also directly stimulate and cause sensitization of their nociceptors (6). Then, enhanced responses of pain circuits in the spinal cord and brain (central sensitization) occurred in the CNS, which takes responsibility for the amplifying signal transmission to the brain and followed alternative in descending pathways (7). Since the periphery and the CNS sensitization is hardly assessable, the level of mediators in the pathway, that is biomarker's levels, have become a standard indicator for evaluating CPSP occurring due to its easy availability. With reference to other clinical studies, we aim to identify specific biomarkers *via* blood sample to predict the occurrence of CPSP (8).

Cytokines involve vitally in the interactions and communications between cells; especially, some are essential in the pathologic process of pain development, thus potentiating chronic pain postoperatively. Multiple studies have identified several traditional cytokines as the risk factors for CPSP such as IL-1β (9) and TNF-α (10). But with the emergence of new neural-inflammatory pathways, the inflammatory mediators’ profiles need to be updated. Recently, C-X-C motif chemokine ligand 10 (CXCL10) (11) levels is found to exacerbates pain in mice. Matrix metalloproteases (MMPs) (12), dedicating to generate neuroinflammation, are implicated in the development of pain. The above cytokines and enzymes hold promise for forming an *in vivo* biomarkers profile to foreshadow the onset of pain. Some clinical variables, such as acute postoperative pain and body mass index (BMI) are also identified as key factors in CPSP predictive model (13). However, previous studies have focused only on the cytokines or the clinical variables separately. A model to predict CPSP occurrence by integration of postoperative inflammatory biomarkers and clinical variables had not been developed.

This is a secondary analysis on follow-up data from a prospective clinical trial, which aim to explore additional options to alleviate MEP after hepatectomy (14). In this study, we hypothesize that novel inflammatory biomarkers play a key role in CPSP development and when combined with clinical variables can predict the onset of CPSP. Specifically, this study aims to explore such a predictive model for CPSP and the clinical utility of machine learning algorithm for modeling. We choose Support Vector Machine (SVM) and random forest due to their different properties.

## Methods

### Participants

In this study (NCT04295330), we performed a secondary analysis on the prospective clinical trials (NCT05492669) in Medical Center: West China Hospital (Sichuan, China) (14) and screened 130 patients suffering from primary liver cancer who underwent elective hepatectomy from 2019 to 2021. Study eligibility included those undergoing elective hepatectomy for primary liver cancer, aged 18–80 years, American Society of Anesthesiologists (ASA) physical status I–III; with follow-up assessment for NRS. All clinical data, including grading of NRS and clinical and physiological parameters were prospectively collected. We excluded patients without follow-up and those body weight <40 or >100 kg; metastases occurring in other distant organs; severe hepatic insufficiency (aspartate aminotransferase or alanine transaminase or bilirubin >2.5 times the upper limit of normal), renal impairment (creatinine clearance <60 ml/min); cardiac rhythm disorders or systolic heart failure (second-and third-degree heart block, ejection fraction <50%); with allergies to any of the trial drugs; chronic opioid use; inability to comprehend numeric rating scale.

### General anesthesia and perioperative procedure

Anesthesia procedures were performed in accordance with standardized criteria. For premedication, patients received 50 mg flurbiprofen axetil by intravenous infusion 30 min before the surgery initiation. Intravenous access to the upper limb was performed and standard monitoring was performed, including electrocardiography, pulse oximetry, blood pressure, and the bispectral index (BIS). Then anesthesia was induced by propofol 1.5–2 mg/kg, midazolam 2 mg and sufentanil 0.2–0.3 μg/kg. Tracheal intubation was facilitated with cis-atracurium 0.2 mg/kg. Whole anesthesia progress was maintained with remifentanil 0.1–0.2 μg/kg/min and desflurane or sevoflurane in a mixture of air 40% and O_2_ 60% to maintain BIS within 40–60. Systolic arterial blood pressure was maintained within 20% of baseline values; when hypotension (MAP <55 mmHg) occurred, patients were treated with intravenous phenylephrine. The incision was infiltrated with 0.25% ropivacaine 20 ml at the end of the procedure. Following the completion of the surgical procedure, the patients were extubated in the operating room and admitted to the post-anesthesia care unit (PACU).

Observations of pain were conducted every 10 min using the numerical rating scale (NRS), and those who scored higher were treated with sufentanil 0.1 µg/kg every 10 min. Aldrete scores were recorded prior to leaving the PACU. It was only possible to discharge patients from the PACU if the Aldrete score was at least 9 and there was no evidence of pain or PONV (15).

Patients received intravenous analgesia with PCIA for the first 72 h post-operatively. In case pain occurs, patient may press the PCIA button repeatedly until feeling relief. Mephedrone 5 mg would be administered if the NRS score of the participant was >3 after taking the maximum dose (10 ml/h).

### NRS and ID pain grading

The day before the operation, all patients received an explanation of how to rate pain intensity on NRS, identifying 0 as “no pain” and 10 as “worst imaginable pain”. In the final assessment of NRS grade, face-to-face follow-ups were conducted 1 day after surgery and telephone follow-ups were conducted 3 months after surgery. Acute postoperative pain is defined as the pain that occurs during movement (e.g., deep breathing) at postoperative 24 h, and CPSP as the pain that occurs at 3 months after surgery.

In the above 3-months follow-ups, the patients suffering from CPSP also received an explanation of ID pain (16). The items included the following: (1) “Did the pain feel like pins and needles?” (2) “Did the pain feel hot/burning?” (3) “Did the pain feel numb?” (4) “Did the pain feel like electrical shocks?” (5) “Is the pain made worse with the touch of clothing or bed sheets?” and (6) “Is the pain limited to your joints?” “Yes” answers to Questions 1–5 were scored as 1, whereas a “yes” answer to Question 6 was scored as −1. Higher scores suggested a neuropathic component to the pain.

### Plasma biomarkers

Blood samples are collected at 24 h after surgery for subsequent measurement, which are labelled, centrifuged, frozen, and stored locally at −80° in line with experimental standards. Levels of 8 plasma biomarkers including VEGF, IL-1β, IL-6, CXCL10, TNF-α, MMP2, MMP3 and MMP9 are measured using Human Premixed Multi-Analyte Kit (R&D, Cat: LXSAHM-15, Lot: L141228).

### Variable selection using random forest

Permuted importance approach in random forest was applied for elimination of some variables that were not predictive for CPSP to improve model accuracy. Permuted importance for a particular variable was calculated by comparing the difference in the prediction accuracy of the out-of-bag data to the prediction accuracy when the variable was noised up by randomly permuting its values. The higher the predictive power variable obtained, the larger importance value it earned. Variables were ranked from high to low according to their permuted importance score and those with the highest scores were selected. Variable selection of this study was performed using R, version 3.6.1.

### CPSP predicting methods

Through the building and cross-validating of predictive models to classify CPSP risk, multiple machine learning algorithms to identify important biomarkers and clinical variables can be employed. After comparison, Support Vector Machines (SVM) and Random Forest were selected. Model building were performed using R, version 3.6.1.

#### SVM

The SVM algorithm was selected considering the limitations of small sample sizes and imbalanced dataset. For SVM model building, limited by the sample size, only 2 features were allowed to avoid over-fitting. The tuning hyperparameters of the SVM included the following: radial, cost, gamma. The SVM classifiers with final features and hyperparameters were trained and tested in Set 1 by 10-times cross-validation (CV) algorithm. SVM models with different sets of features and sets of hyperparameters were compared by AUC; finally, the one with the highest value was selected as the final SVM classifier. In the process of constructing the SVM model, we set radical as kernel, cost as 20 and gamma as 0.01 by the result of automatic parameter tuning. The generalized performances of this final predicting classifier were validated in Set 2, including accuracy, sensitivity, specificity receiver operative curves (ROCs), and its corresponding AUC.

#### Random forest

Random forest was selected for its excellent performance on handling an imbalanced dataset containing multiple types of data. Multiple decision trees were built based on bootstrap sampling of the Set 1 by 10-times cross-validation (CV) algorithm. It could give a prediction whether a patient would develop CPSP by providing a probability, and the probability was determined by the ratio of the decision trees that gave positive results to the total number of decision trees. In the process of constructing the RF model, we set ntrees as 280 and mtry as 3 by the result of manual parameter tuning, considering the predictive performance of the RF model and AUC. The generalized performances of this final predicting classifier were also validated in Set 2 as previously mentioned.

## Results

### Recruitment

**Figure 1:**
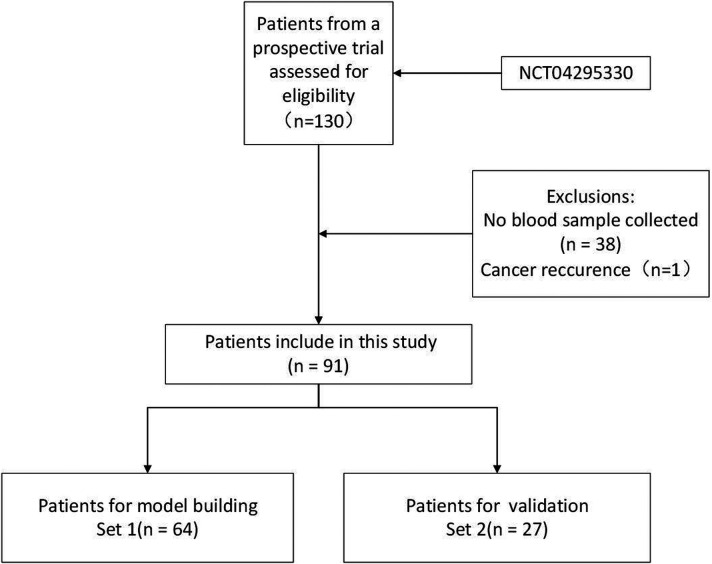
Study flow chart.

Participants were selected from a clinical trial recruiting between June 2019 and June 2021. Participant flow through the study was shown in Figure 1. After the initial screening, 130 patients were deemed eligible to participate in the study. Participants with missing data were removed. Of these, 91 (70.0%) patients completed the blood sample collected and follow-up at 3 months, while radiological examinations were performed to exclude recurrence of the primary tumor, distant metastases from the primary tumor, and new tumors. Thus, this report presents results of 91 participants who were included in the final analysis.

### Participant characteristics

91 individuals undergoing hepatectomy were included in the analyses. 70 (77.0%) were male; 21 (23.0%) were female. The mean ± SD. age of all participants was 54.1 ± 11.0 years (range 27–78 years). The average duration of surgery was 3.2 ± 1.2 h, and the average duration of anesthesia was 4.4 ± 1.2 h. The average volume of bleeding was 289.8 ml, which varying from 10 to 1,200 ml. Open hepatectomy was performed in 76 patients (83.51%), while laparoscopic hepatectomy was performed in 15 patients (16.49%). Of these, the incision length varied from 23 to 30 cm for open procedures, with an average of 26.82 cm.

All participants reported having acute postoperative pain. Of these, 33 (36.2%) reported mild pain (NRS 1–3), 46 (50.6%) reported moderate pain (NRS 4–6), and 12 (13.2%) reported severe pain (NRS > 6).

**Figure 2:**
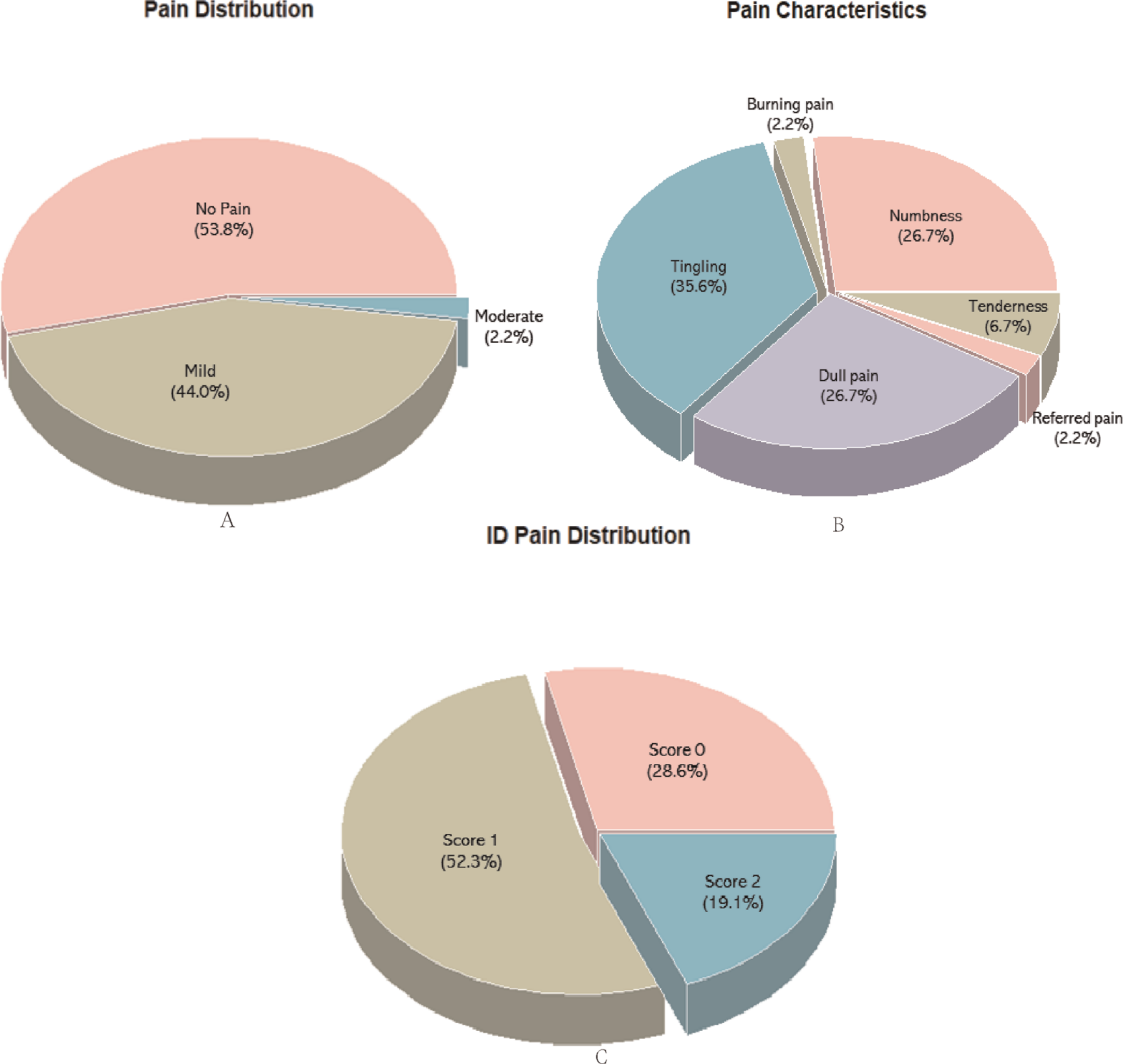
NRS (**A**), pain characteristics (**B**) and ID pain (**C**) at 3 months postoperatively.

There were 49 (53.8%) participants who reported no pain 3 months after surgery, 40 (44.0%) participants who reported mild pain, and 2 (2.2%) participants who reported moderate pain. Pain distribution and characteristics were shown in Figures 2A,B.

More recently, the six-item ID Pain was developed as a brief, self-administered screening tool for detecting neuropathic pain in primary care settings (16). Among those who reported CPSP, 12 scored 0 (28.6%), indicating they were less likely to be neuropathic; while 22 scored 1 (52.3%) and 8 scored 2 (19.1%), indicating that most patients suffer from CPSP associated with neuropathic pain. The results of the ID pain distribution were shown in Figure 2C.

### Univariate analysis of biomarkers and clinical variables

**Figure 3:**
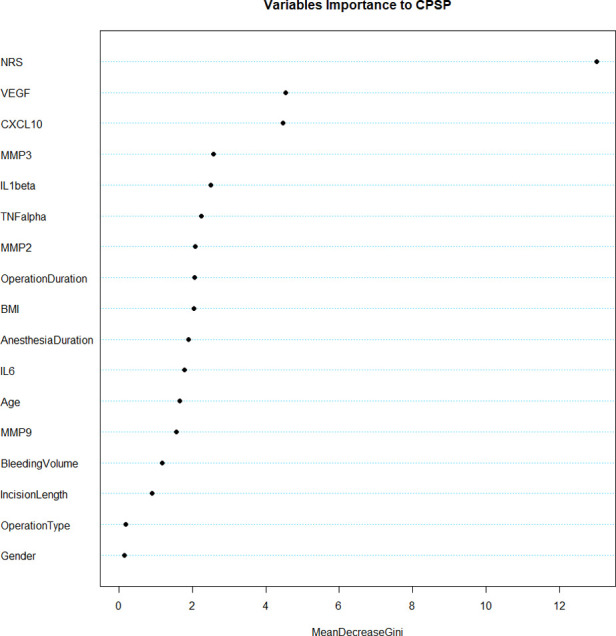
Random forest analysis of clinical variables and biomarkers.

To explore the effect of each biomarker and clinical variables, random forest was performed for them shown in Figure 3. NRS (13.14) was signiﬁcantly associated with the risk of CPSP. Also, CXCL10 (4.24), MMP3 (2.53), TNF-α (2.10), IL-1β (2.27), MMP2 (2.14), Anesthesia Duration (2.20), Operation Duration (2.22) and VEGF (4.96) were also signiﬁcantly related to the risk of CPSP (threshold = 2).

### Models building

91 patients were randomized into two independent sets: 64 patients for model building and cross validation in train set (Set 1), and 27 patients in test set (Set 2). Two groups share similar physical and clinical characteristics.

**Figure 4:**
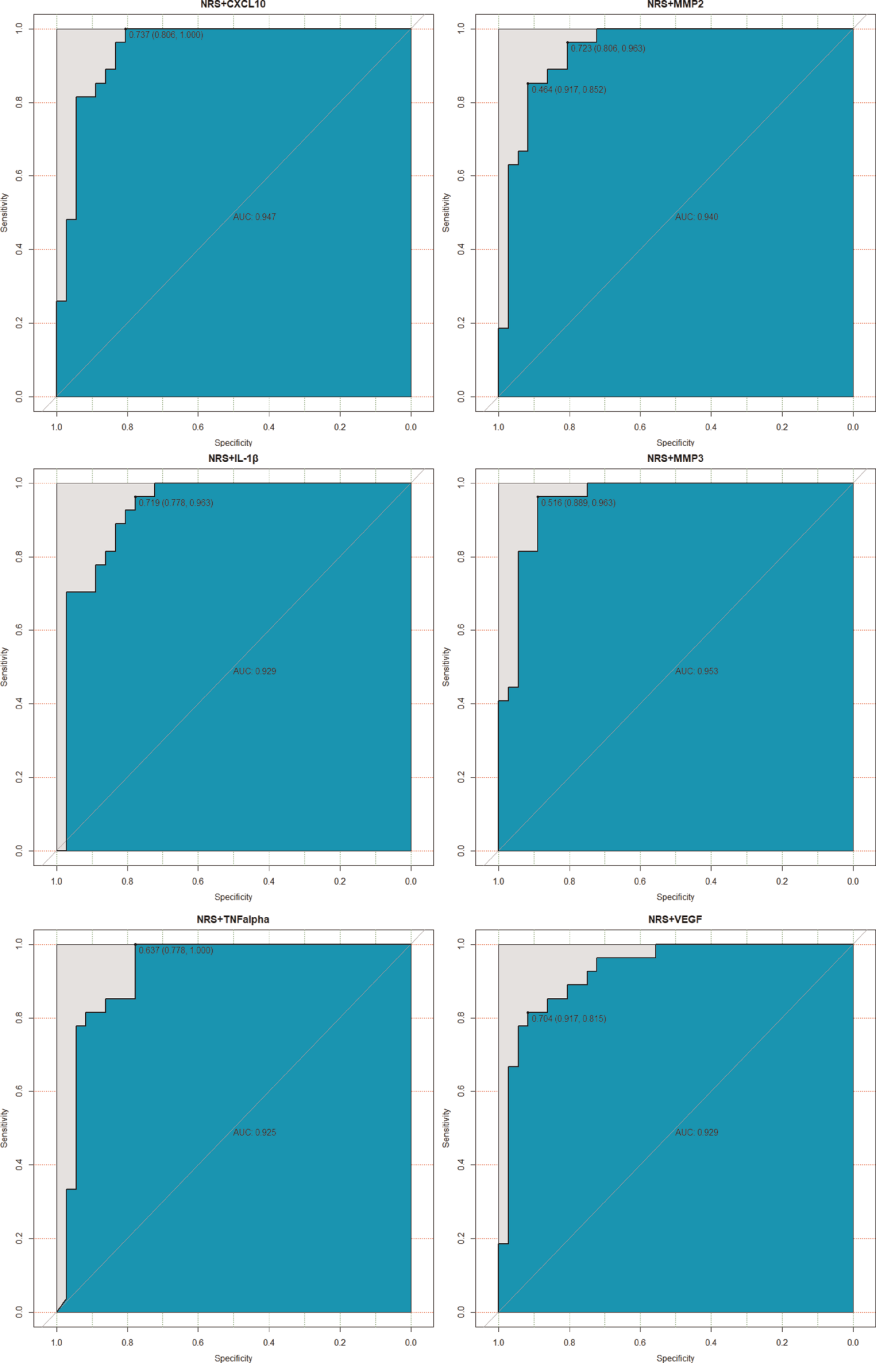
Performances of the ﬁnal SVM model (NRS + MMP3) in Set 1 comparing with the other model. The panel showed their ROC performances. The models’ features were shown in subplots’ head. The panel demonstrated that the ﬁnal model of using NRS and MMP3 with the best performance. ROC, receiver operating characteristic; AUC, the area under the ROC curve.

**Figure 5:**
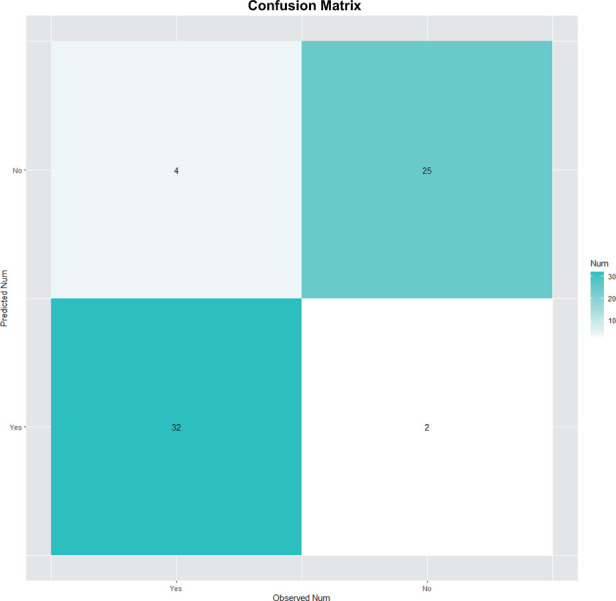
Predicted result of the ﬁnal SVM model for CPSP risk in Set 1.

SVM model for CPSP risk were ﬁrst trained and tested in Set1 by cross validation. The model with best performance was selected, and this model included 2 features from biomarkers and clinical variables of our interest. NRS at the first postoperative day seemed to be the most signiﬁcant one as identiﬁed by random forest shown in Figure 3. In line with this, previous studies attached great importance to postoperative acute pain towards the onset and progress of CPSP (17, 18). As mentioned above, we finally picked it as a representative clinical variable. CXCL10, MMP3, TNF-α, IL-1β, MMP2 and VEGF were also taken into account in this model not only for its remarkable importance, but for indispensable role in neuro-inflammatory pathways. For better visual comparison, models including CXCL10, MMP3, VEGF, TNF-α, IL-1β, MMP2 and NRS were shown in Figure 4 to compare their performances. The ﬁnal SVM model with radial kernel included NRS and MMP3 as model features, while cost = 2, gamma = 0.01 as model parameters. The classifying performance measures in Set 1 included: accuracy = 0.890, sensitivity = 0.963, speciﬁcity = 0.889, AUC = 0.953. Figure 5 showed the predicted results of this ﬁnal SVM model for Set 1 patients, 32/36 patients actually had CPSP when they were predicted to have CPSP, while 25/27 patients without CPSP were predicted to not develop CPSP. Matrix metalloproteinases (MMPs) have been implicated in the modulation of synaptic plasticity, glial activation, and long-term potentiation in the CNS and MMP3 was proved to regulate of nociceptive processing in thermal hyperalgesia and tactile hypersensitivity (19), which was consistent with the ID pain result above that most CPSP associated with neuropathic pain.

**Figure 6:**
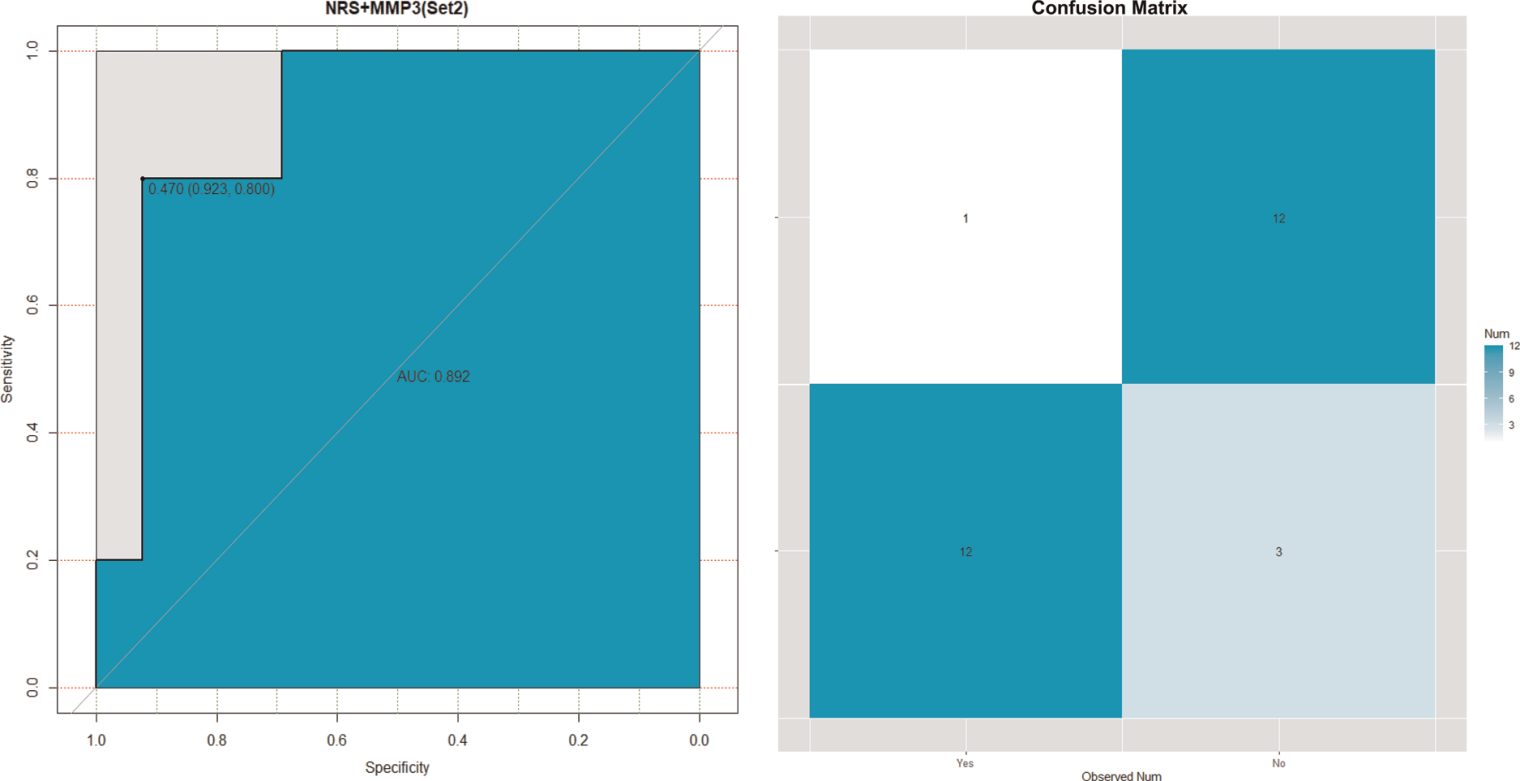
Roc curve and predicted result of the ﬁnal SVM model for CPSP risk in Set 2.

The ﬁnal SVM model was validated in Set 2. It predicted CPSP risk with accuracy = 0.857, sensitivity = 0.800, speciﬁcity = 0.923, AUC = 0.892. The validated results are shown in Figure 6: 12/15 patients actually had CPSP when they were predicted to have CPSP, while 12/13 patients without CPSP were predicted to not develop CPSP.

**Figure 7:**
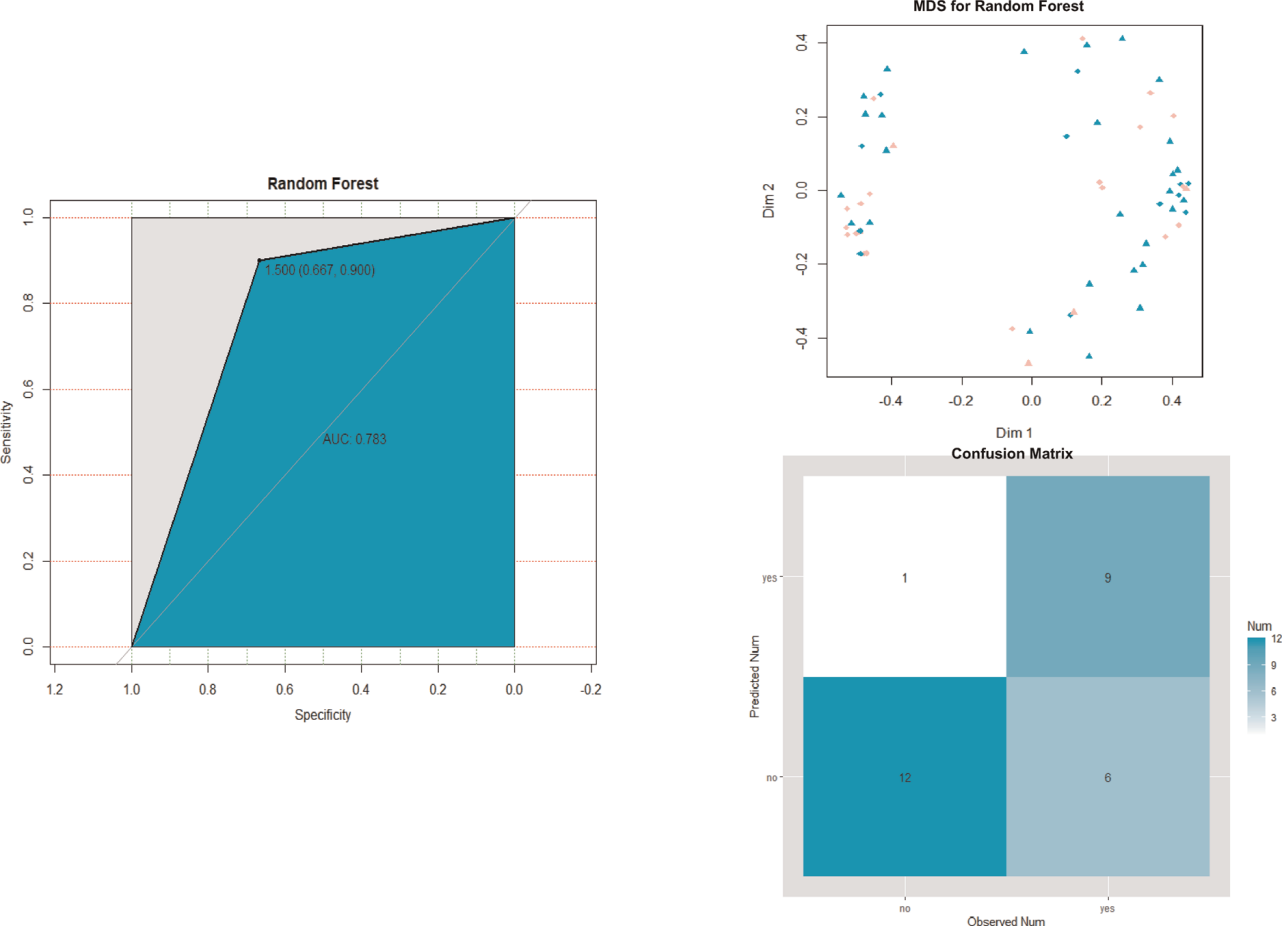
Roc curve, MDS plot and predicted result of the ﬁnal random forest model for CPSP risk in Set 2. MDS, multidimensional scaling.

The performances of the ﬁnal SVM classiﬁer were compared with that of random forest model (also built in Set 1 and tested in Set 2). As shown in Figure 7, the ROC curve (AUC = 0.917) of the ﬁnal SVM classiﬁer was not only higher than that of the SVM classiﬁers with other features of our interest as described above Set 1, but also higher than random forest model (AUC = 0.783).

## Discussion

In the current study, the overall prevalence of CPSP at 3 months after hepatectomy was 53.85% (49 in 91). Findings from this study proved that the inflammatory biomarkers MMP3 and NRS at 24 h postoperatively correlated significantly with the risk of CPSP. Machine Learning model combining MMP3 and NRS for CPSP risk forecasting showed reasonable predictive values. Within the limitations of a medium-sized study, the AUC of the final SVM model showed sound performance for predicting CPSP risk.

Previous studies had underscored that learning machine methods could better predict the occurrence of multiple diseases (20, 21) due to high model accuracy and reduced overfitting. SVMs were highly resistant to overfitting as they map to a finite dimensional space (22). In line with this, our data (Figures 5–7) showed that models generated by support vector machines have better performance than random forest models. Furthermore, as shown in Figures 5, 6, the support vector machine model could be used as an intuitive and convenient tool to facilitate clinical decision-making. For example, the anesthetist could estimate the risk of CPSP by assessing the baseline level of MMP3 and the 24 h postoperative NRS. If a patient with an estimated high risk of CPSP, the anesthetist was able to modify the perioperative pain administration to reduce the CPSP risk.

SVM was considered a “black box” algorithm, which might create a challenging dilemma for those responsible for a potential decision-making medical error. Interpreting how the model reached its predictions was therefore necessary to enable researchers to assess whether their predictions were trustworthy (23). Accumulating evidence have identified several risk factors for CPSP, including presence and intensity of acute postoperative pain, type of surgery, age, body mass index (BMI) and psychosocial factors (24, 25). Here, we proved NRS at postoperative 24 h to be an essential part to predict CPSP (Figure 3), that is to say, postoperative acute pain might lead to the development of chronic pain. Multiple studies have backed this perspective (26, 27) and the involving mechanism might include neuroplasticity, pain modulation and central sensitization (28). Therefore, this factor was first included in model building. Notably, in this study, blood samples were only collected from patients 24 h after surgery, and it was found to be strongly correlated with the development of CPSP. However, a few studies did not support the direct transition from acute nociceptive postoperative pain to a different chronic pain condition as a mechanism for CPSP, but rather the parallel development of different types of persistent pain, including some perioperative or postoperative events, with the remission of acute pain (29). Due to the limitations of the data, we considered acute postoperative pain to be a highly potent risk factor, but the role of perioperative or postoperative events 3 months after surgery could not be dismissed. Few studies have explored the predictive models of inflammatory cytokines for CPSP, but most focused on local inflammation, with very few focusing on neuro-inflammatory conditions. We took a stab at building predictive models with novel biomarker profile at 24 h postoperative levels covering both above and were surprised to find that the model including MMP3 performed remarkably well (Figures 4–6). Present studies showed MMP3 to be a key component concurrent with increased tumor necrosis factor (TNF) in a spinal cascade initiated by peripheral inflammation and resulting in thermal hyperalgesia and tactile allodynia, likely from non-neuronal cells to evoke a facilitated state of dorsal horn processing (19). For clinic, elevated MMP3 has been reported in patients with ankylosing spondylitis, a chronic inflammatory disease, compared with healthy controls (30). Also, a correlation between MMP-3 and IL-6 was proposed in fibromyalgia, a common chronic pain, which represents either inflammatory cytokine-induced MMP-3 release or MMP-3 stimulation of local inflammatory cytokine production (31).

Meanwhile, this study also demonstrates the importance of other biomarkers (e.g., CXCL10) and clinical variables (e.g., operation duration, anesthesia duration and BMI) in patients after hepatectomy, although they were not entered into the SVM model implemented by the machine learning algorithm. In mice with spinal nerve ligation (SNL), CXCL10-activated CXCR3 was found to mediate p38 and ERK activation in DRG neurons and enhance neuronal excitability, which contributed to the maintenance of neuropathic pain (11). BMI was also reported to be a crucial risk factor for CPSP in cardiac surgery because overweighting or obesity increased the technical difficulty and might expose patients to prolonged retraction giving rise to CPSP (32). However, on account of the small database, the model did not incorporate the above variables to avoid overfitting. Future studies are warranted to determine the additional contribution of CXCL10 and BMI to CPSP as well as to externally validate our results.

There were some limitations of this study. First, the sample size was small limiting statistical evaluation and constraining our choice in machine learning model; secondly, only 2 factors were applied to construct the SVM model to avoid overfitting but was susceptible to type I error and unfitting; additionally, limited by the numbers of the events, this model simplified CPSP as a binary outcome, while the NRS was a 10-level graded event and pain also varied in character, which were topics of our ongoing study; what's more, only biomarkers that have been widely studied recently were included in this study, and other biomarkers were also worthy of further exploration. These limitations could be overcome in future with larger sample sizes.

In summary, using a machine learning framework, a SVM model of CPSP risk was established which integrated MMP3 and NRS as representative of cytokines and clinical variables. The SVM classiﬁer was validated in Set 2 and conﬁrmed to have reliable predictive performance. Additionally, our study provided important insights into biomarkers of CPSP risk and had identiﬁed 24 h postoperative biomarker levels such as VEGF, MMP3 and CXCL10 and clinical variables to be closely related to CPSP. Further study will need to validate this ﬁnding and will need to consider the incorporation of more biomarkers and clinical variables to improve predictive accuracy.

## Data Availability

The raw data supporting the conclusions of this article will be made available by the authors, without undue reservation.
